# In vitro antimicrobial effect and mechanism of action of plasma-activated liquid on planktonic *Neisseria gonorrhoeae*

**DOI:** 10.1080/21655979.2021.1955548

**Published:** 2021-07-29

**Authors:** Jia Liu, Chunjun Yang, Cheng Cheng, Chenchen Zhang, Jun Zhao, Chuyu Fu

**Affiliations:** a Department of Dermatology, the Second Affiliated Hospital, Anhui Medical University, Hefei, People’s Republic of China; b Institute of Plasma Physics, Chinese Academy of Sciences, Hefei, People’s Republic of China

**Keywords:** *Neisseria gonorrhoeae*, plasma-activated liquid, biocidal efficacy, antimicrobial mechanism, inflammation

## Abstract

*Neisseria gonorrhoeae* (*Ng*) is highly resistant to treatment, and there is an urgent need for new treatments to alleviate gonococcal resistance caused by antibiotic monotherapy. The antimicrobial effect and mechanism of plasma-activated liquid (PAL) on *Ng* were evaluated in this study. Upon PAL treatment, extensively analyses on cell culturability, metabolic capacity, intracellular reactive oxygen species (ROS),membrane integrity and nucleic acids for Ng were carried out and significant antimicrobial effects observed.PAL exerted antibacterial effect on *Ng* and induced bacterial death (6.71-log) following immersion for 30 min and treatment for 120 s. However, bacterial viability test revealed that after immersion in the same PAL, 10.17% of bacteria retained their metabolic capacity. This indicates that bacteria enter a physiological viable but non-culturable state to protect themselves from environmental stress. Confocal fluorescence microscopy and transmission electron microscopy demonstrated that PAL exerts bactericidal effect on *Ng* and disrupts its morphological structure. PAL may upregulate inflammatory factors and genes to modulate the resistance of *Ng* and affect the immune status of the host during infection.

## GRAPHICAL ABSTRACT

**Figure:**
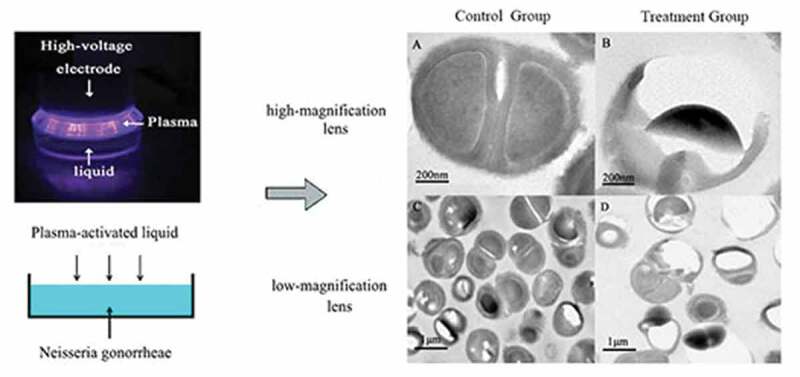

## Introduction

*Neisseria gonorrhoeae* (*Ng*) is a gram-negative diplococcus bacterium that primarily invades the mucosal tissues, and its infection is the second most common sexually transmitted disease in China[1]. Neonatal gonococcal conjunctivitis is a highly infectious and destructive acute purulent conjunctivitis caused by *Ng*; it leads to corneal ulcers, perforation, and blindness. Furthermore, *Ng* facilitates HIV transmission, causing high morbidity and increasing socioeconomic consequences[2]. Gonorrhea generally manifests as an acute purulent genital tract infection by *Ng* and is characterized by abundant activated neutrophils and exudates[2]. In most countries, antibiotics are the recommended first-line treatment for gonorrhea[3]. However, owing to the abuse of antibiotics, antimicrobial resistance in *Ng* has become a serious threat to global public health[4]. Furthermore, *Ng* is a highly adaptable pathogen that can evade the host’s innate immune response and suppress adaptive immunity[5], posing a substantial challenge in gonorrhea treatment and control.

Plasma, a neutral ionized gas that can be generated under natural or artificial conditions, comprises various particles produced by gas ionization under heating or strong electromagnetic fields[6]. Plasma-activated liquid (PAL) is generated from the interaction between atmospheric plasma and liquid (physiological saline). Recently, PAL has garnered considerable attention as it is reportedly effective against a wide range of microorganisms, including bacteria and yeast, as well as biofilms and spores [7–10]. Compared with antibiotics, PAL sterilization has the advantage of high efficiency [11,12]. Physiological saline is the most commonly used isotonic solution in clinical settings, and its application simulates the body fluid environment better than other media.

In this study, we aimed to evaluate the antimicrobial effect and the mechanism of action of PAL against *Ng*. For this purpose, we examined the ability of PAL to inactivate *Ng* and the subsequent changes in cell morphology and functioning, including cell membrane integrity, cell metabolism, and intracellular reactive oxygen species (ROS) levels. We also evaluated the effect of PAL on inflammation.

## Material and methods

### Animals and strains

A strain of *Ng* (BNCC337543) was purchased from the BeNe Culture collection. Male BALB/c mice (aged 6–8 weeks; 18–20 g) were obtained from the Anhui Medical University Laboratory Animal Center. All animal studies were reviewed and approved by the Biomedical Ethics Committee of Anhui Medical University (20,200,355).

### Atmospheric pressure air plasma

**Figure 1.**
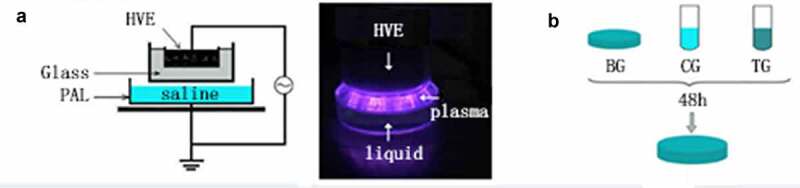
(a) Diagram of plasma device and schematic diagram of plasma-activated liquid (PAL) treatment of *Neisseria gonorrhoeae*. Experimental schematic of the blank, control, and treatment groups. The blank group is the untreated group. Figure 1(b) In the control and treatment groups, the bacteria were immersed for 30 min in physiological saline and PAL, respectively

**Figure 2.**
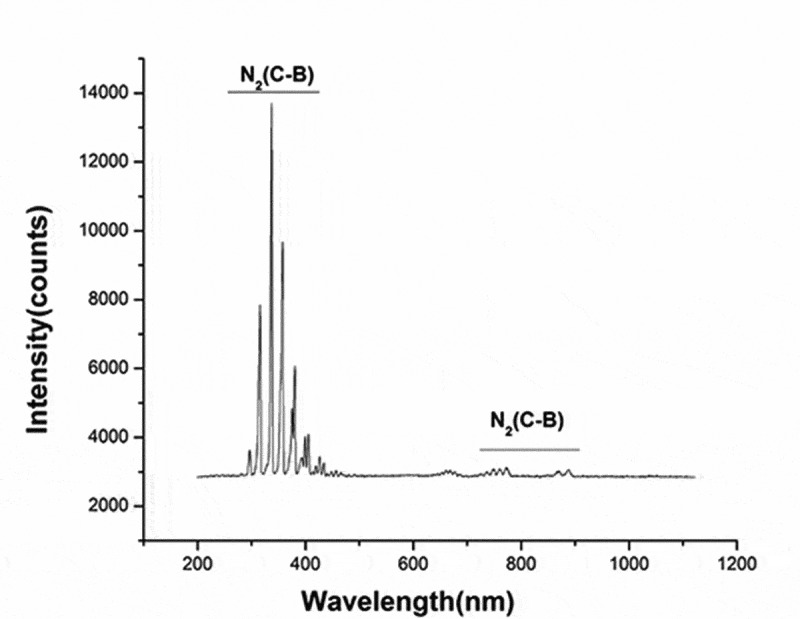
Voltage and current waveforms of atmospheric pressure plasma

**Figure 3.**
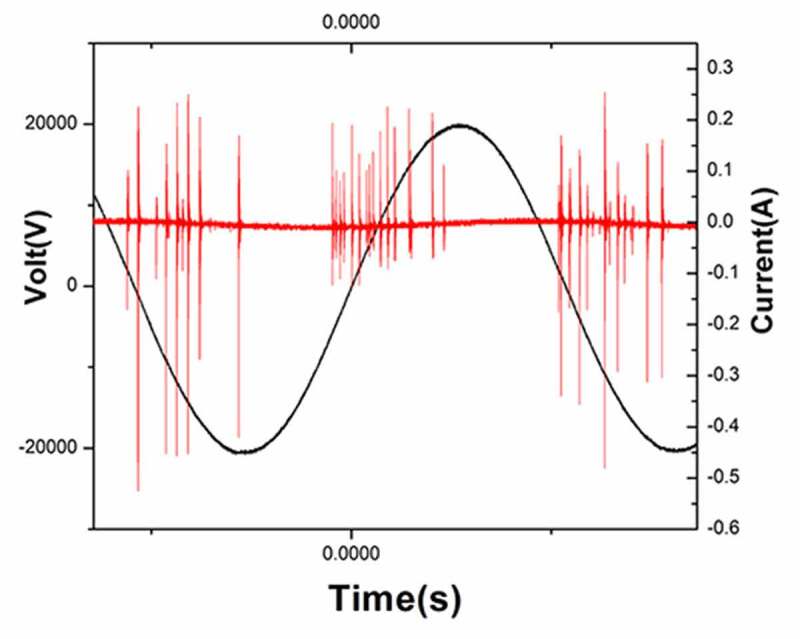
Spectrum, current, and voltage signal diagram of plasma center point under specific parameters

The atmospheric pressure plasma equipment that we used to prepare PAL is shown in Figure 1. It is composed of a high-voltage electrode, ground electrode, and power source. A dish was placed between the two electrodes, and 3 mL of physiological saline was injected into the dish. The discharge interval between the surface of the liquid and the bottom of the quartz glass was fixed at 4 mm. Atmospheric pressure plasma was applied to physiological saline for different periods to prepare PAL. The bacteria were then immersed in PAL (Figure 1a). A Tektronix MSO 5104 (Tektronix, Shanghai, China) digital oscilloscope, 1000:1 high-voltage probe (Tektronix P6015A; Tektronix), and current probe (Tektronix P6021; Tektronix) were used to monitor the voltage and current applied to the atmospheric pressure plasma (Figure 2). The optical emission spectra of plasma were detected using a spectrometer (Figure 3)[13].

### Bacterial strains and culture conditions

The *Ng* strain was reconstituted from frozen stock cultures and propagated at 37°C with 5% CO_2_ on GC agar (OXOID, Basingstoke, UK). Before each treatment, the bacterial concentration was adjusted to 5 × 10^6^ CFU mL^−1^ (colony-forming units per milliliter)[14].

### Inactivation of Ng blank, control, and treatment groups

Physiological saline was treated with plasma for 60 s to prepare PAL. One hundred microliters of bacterial suspension was used in each experiment, and 900 µL of PAL/physiological saline was used as the reaction solution. Bacteria were immersed in the reaction solution for 30 min (Figure 1b). After culturing for 48 h, *Ng* colonies were counted. The untreated group served as the blank group and bacteria immersed in physiological saline for 30 min were used as the control group.

### Inactivation of Ng by PAL

Physiological saline was treated with plasma for 10, 30, 60, 90, and 120 s to prepare PAL. The bacteria were immersed in PAL for different periods (1, 10, and 30 min). After culturing for 48 h, the survival rate of bacteria was calculated, and the cells were immersed in physiological saline for the same periods as the control group.

### Bacterial viability test assay

Resazurin (Sigma Aldrich, USA) was used to conduct in vitro toxicity test using the antibiotic susceptibility (mg^−^[1]) method to detect the metabolic activity of bacteria[15]. After immersing the bacterial cells in PAL for 30 min, the supernatant was removed and cultured with resazurin. The samples were analyzed using a microplate reader (BioTeK, VT, USA) at excitation/emission wavelengths of 560/590 nm.

### Intracellular ROS concentration

The intracellular ROS concentration was evaluated using a ROS assay kit (Beyotime, China)[16]. After immersing the bacterial cells in PAL for 30 min, the bacterial suspension was mixed with diluted 2,7-dichlorodihydrofluorescein diacetate (DCFH-DA) solution and incubated for 20 min at 37°C in the dark. Samples were analyzed using a microplate reader with excitation/emission wavelengths of 488/525 nm.

### Reactive species in PAL

Using a spectrophotometer and PhotoLab 6100 (WTW), along with test kits, the concentration of reactive species (RS) in PAL, including H_2_O_2_, NO_3_^−^, and PH, was evaluated[17].

### Analysis of membrane integrity

The LIVE/DEAD®BacLight™ bacterial viability kit (Invitrogen, USA) was used to determine membrane integrity. Saline was treated with plasma for different periods, and then bacteria were immersed in PAL for 30 min. The bacteria were stained with the reagent available in the bacterial viability kit and incubated at 37°C for 15–20 min. Next, a fluorescence microscope was used to analyze the samples[18].

### Electron microscopy examination of plasma-treated cells

Physiological saline was exposed to the plasma for 60 s; thereafter, the bacteria were immersed in PAL/physiological saline for 30 min, with the physiological saline-treated bacteria as the control group. The bacteria were incubated in 3% glutaraldehyde and fixed overnight. Subsequently, 1% osmium tetroxide was added to the solution for 90 min. The samples were embedded in epoxy resin overnight. The sections were stained and examined by transmission electron microscopy (TEM).

### Fluorescence quantitative PCR detection of bacterial colonization-related genes

The bacterial cells were immersed in PAL for 30 min, and the solution was centrifuged to remove the supernatant. The bacterial cells were then cultured for 6 h, and then SYBR Green nucleic acid dye (Takara, Japan) was used as a fluorescent probe and fluorescent quantitative PCR (qPCR) was performed to detect the expression of *OxyR* and *PliC1* in the cells. The forward (F) and reverse (R) primer sequences used for qPCR are presented in Table 1.

**Table 1.** Primer sequences used to detect *PliC1* and *OxyR.*

Gene	Primer sequence	Expected product size
16S RNA	F: 5ʹ-TATCGGAACGTACCGGGTAGC-3’	426 bp
16S RNA	R: 5ʹ-GTATTACCGCGGCTGCTGGCA-3’	
*OxyR*	F: 5ʹ-TCAGCCAGCCCACTTTGTCTA-3’	150 bp
*OxyR*	R: 5ʹ-GCTCCGCCTCTTTCAATACCTT-3’	
*PliC1*	F: 5ʹ-ATGCGGGCAGGCTGAATAG-3’	223 bp
*PliC1*	R: 5ʹ-CAGTCGCCAAGGGAAATACG-3’	

### Murine splenocytes infection model

The mice were anesthetized with 2% pentobarbital, and the spleen was aseptically removed and homogenized. Spleen cells were separated. An *Ng* suspension (5–10 × 10^6^ CFU) was prepared, and physiological saline was exposed to plasma for 60 s to obtain PAL. The mouse nuclear cells were divided into four experimental groups: (1) blank control group (cells were immersed in PBS solution for 30 min); (2) plasma-activated mouse splenocyte group (mouse splenocytes were immersed in PAL for 30 min); (3) *Ng-* and plasma-activated mouse splenocyte co-treatment group (mouse splenocytes were exposed to *Ng* suspension and PAL for 30 min); and (4) simple gonococcal infection group (mouse splenocytes were incubated with *Ng* suspension for 30 min). The groups were then placed in a 37°C, 5% CO_2_ incubator for 6 h, and subjected to qPCR to detect *IL-1β* expression using the primers presented in Table 2. Moreover, ELISA (Abcam, UK) was performed to detect IL-10 and IL-17 levels [19] in cell culture supernatants.

**Table 2.** Primer sequences used to detect *IL-1β.*

Gene	Primer sequence	Expected product size
*Gapdh*	F: 5ʹ-TAAAAGCAGCCCTGGTGACC-3’	426 bp
*Gapdh*	R: 5ʹ-CCACATCGCTCAGACACCAT-3’	
*IL-1β*	F: 5ʹ-CTGCTGGTGTGTGACGTTCC-3’	426 bp
*IL-1β*	R: 5ʹ-ATATGGGTCCGACAGCACGA-3’	

### Statistical analysis

The analysis of variance and *t*-test were performed using IBM SPSS Statistics 21 (IBM Corp. Armonk, NY, USA) to evaluate the differences between experimental groups. Results were considered statistically significant at *P <* 0.05.

## Results

In this research, the antimicrobial effects and mechanisms of PAL on Ng were studied and the effects of PAL treatment on the expression of inflammatory genes and factors were examined in a mouse spleen cell monocyte infection model. CFU and bacterial viability tests demonstrated that PAL exerts a clear bactericidal effect on *Ng*. Confocal fluorescence microscopy and TEM showed that the cell structure of Ng was destroyed. Furthermore, PAL disrupted bacterial pili-related gene PliC1 and reactive oxygen species-related gene OxyR expression. Moreover, in the in vitro mouse infection model, the expression levels of inflammatory genes and factors increased rapidly after treatment with PAL.

### Discharge waveform and emission spectrum of plasma

The discharge voltage and discharge current of gas-liquid plasma were 20 kV and 400 mA, respectively (the waveform diagram is shown in Figure 2). The optical emission spectrum of plasma under the experimental parameters is shown in Figure 3. The intense emissions from N_2_(C-B) and the second order emission from N_2_(C-B) were observed between 311–317 and 600–800 nm, respectively.

### Inactivation of Ng in the blank, control, and treatment groups

**Figure 4.**
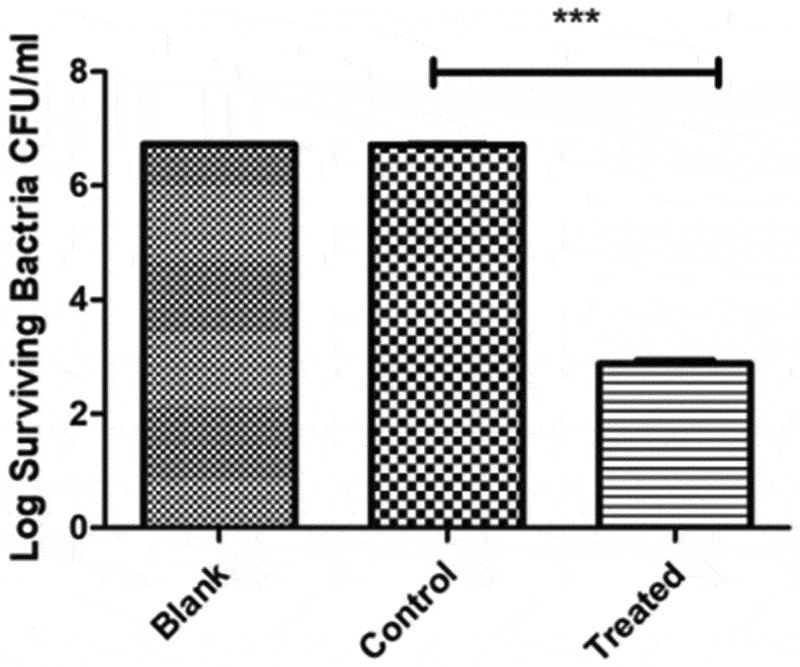
Comparison of plate count results between the treatment, blank, and control groups. No difference was found between the blank and control groups (*P* = 0.662, *P* > 0.05). A significant difference was found between the blank and control groups (*P* < 0.0001)

The blank group was the untreated group, and bacteria were immersed in physiological saline for 30 min in the control group (Figure 4). Bacteria were immersed in PAL for 30 min in the treatment group. After a 48-h incubation period, the number of *Ng* cells was in an order of magnitude of 6.72-log CFU mL^−1^ in the blank group and 6.72-log in the control group. No difference was observed between the blank and control groups (*P* = 0.662, *P* > 0.05). The number of bacteria was approximately 2.88-log CFU mL^−1^ in the treatment group, significantly different from that in the control group (*P* < 0.0001).

### Time- and dose-dependent response of Ng to PAL treatment

**Figure 5.**
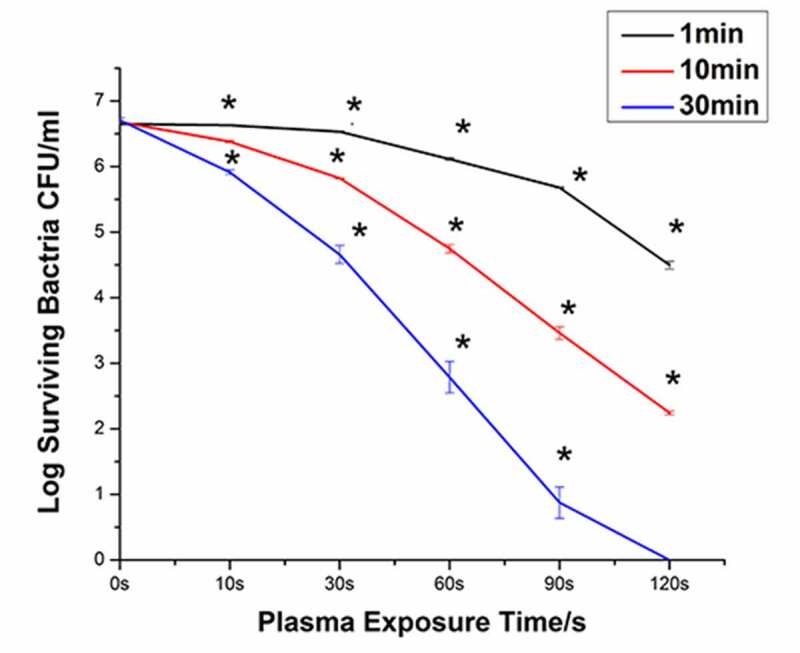
Destruction curves of Neisseria gonorrhoeae strains at different discharge times and under different plasma-activated liquid (PAL) immersion periods (P < 0.05)

Figure 5 shows the bacterial survival curves generated following immersion in PAL for 1, 10, and 30 min. When the plasma treatment time was 10 s and the immersion time was 1 min, the bacteria were killed by 0.03 orders of magnitude. When the plasma treatment time was 120 s and the immersion time was 1 min, the number of bacteria decreased from 6.65^−log^ to 4.50^−log^. The bactericidal efficiency of PAL increased with the increase in the plasma treatment time. When the immersion time was extended to 30 min and the plasma treatment time was 120 s, all bacteria were killed (6.71-log). The bactericidal efficiency of PAL increased with the increase in the immersion time.

### Bacterial metabolic activity

**Figure 6.**
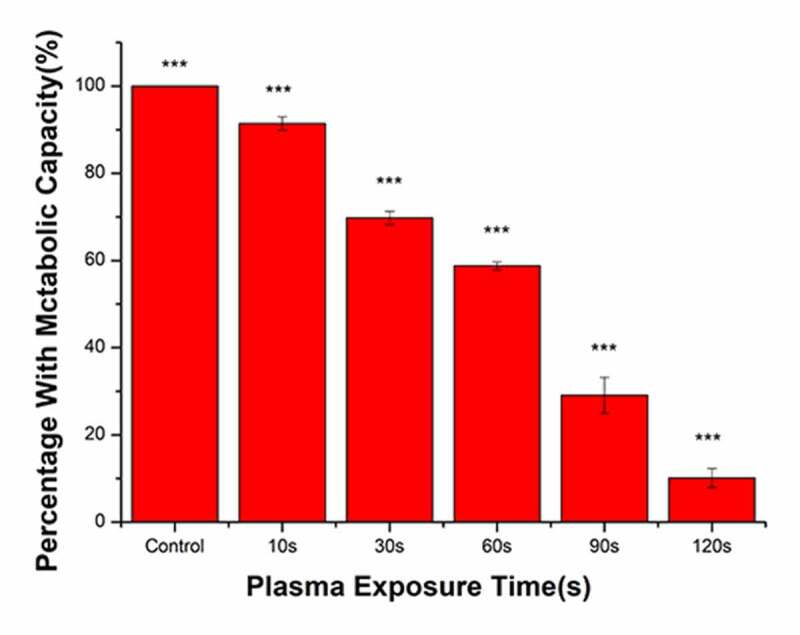
Changes in the metabolic capacity of bacteria immersed in plasma-activated liquid (PAL) for different times (F = 806.5, *P* < 0.0001)

The metabolic activity of bacteria in the control group was set at 100%, and corresponding metabolic activity ratios in the treatment groups were calculated (Figure 6). With the increase in the plasma treatment time, the metabolic activity of bacteria gradually decreased. The plasma treatment time was 120 s and the proportion of metabolically active bacteria was 10.17%. Compared with that in the control group, the metabolic activity of bacteria was significantly reduced following treatment with PAL.

### ROS detection in bacteria

**Figure 7.**
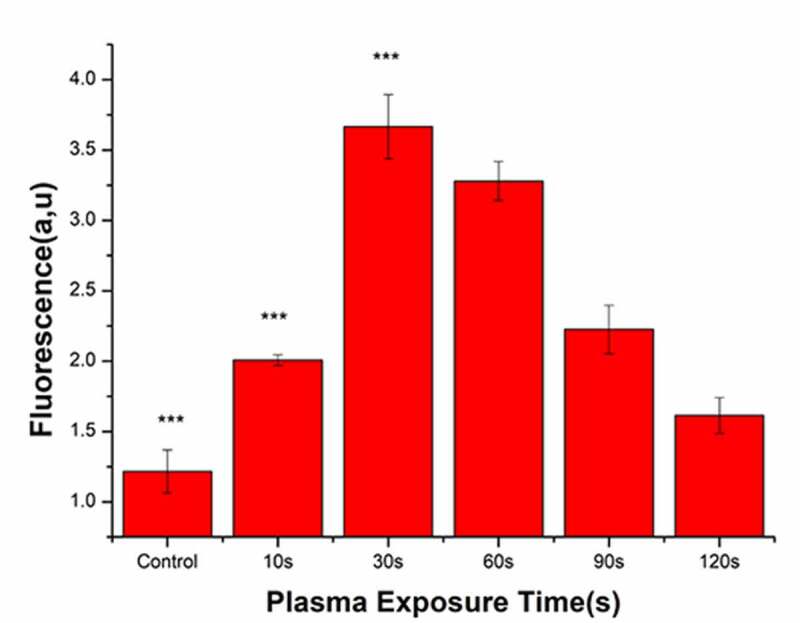
Reactive oxygen species (ROS) concentrations in bacteria after treatment with the plasma-activated liquid (PAL) (F = 214, P < 0.05)

Following plasma treatment, the concentration of ROS in the bacterial cells initially increased and subsequently decreased, with a peak value at 30 s (Figure 7). After treatment with PAL, the concentration of ROS in the bacteria increased from 1.42 to 3.58 at 30 s. Overall, ROS generation in the bacteria gradually decreased from 60 to 120 s.

### Active substance detection in PAL

**Figure 8.**
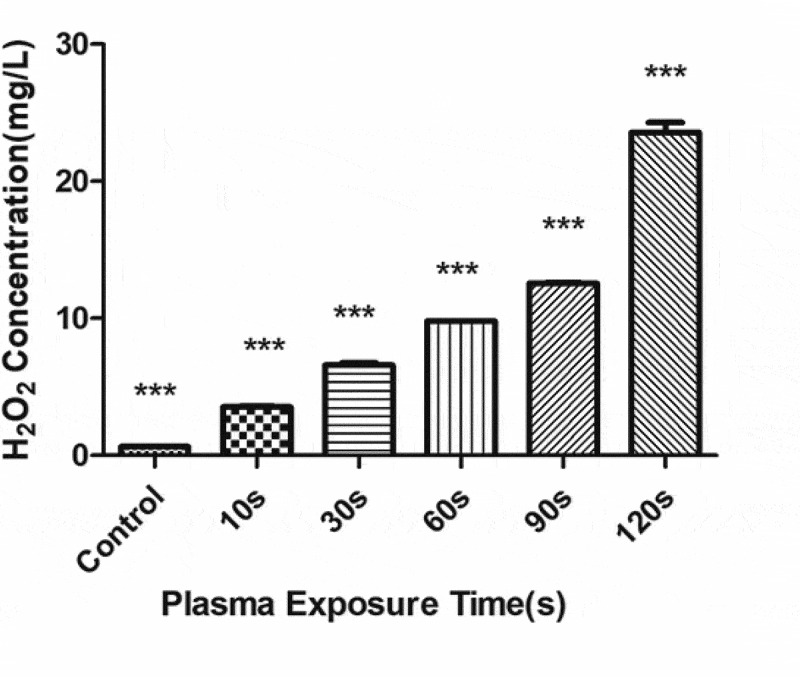
Changes in the H_2_O_2_ concentration in the plasma-activated liquid (PAL) treated for different periods (F = 718.5, *P* < 0.01)

**Figure 9.**
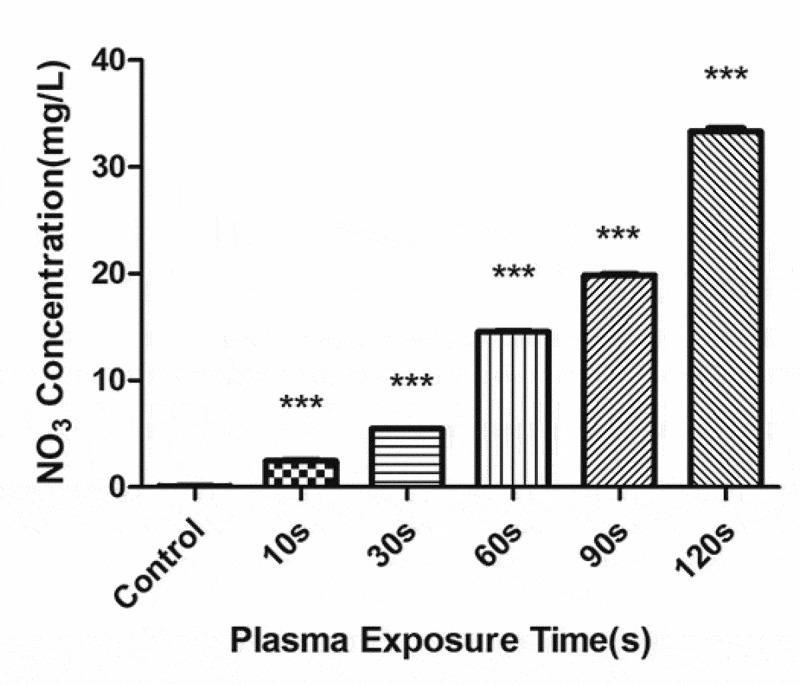
Changes in the NO_3_ concentration in the plasma-activated liquid (PAL) treated for different periods (F = 7228, *P* < 0.01)

**Figure 10.**
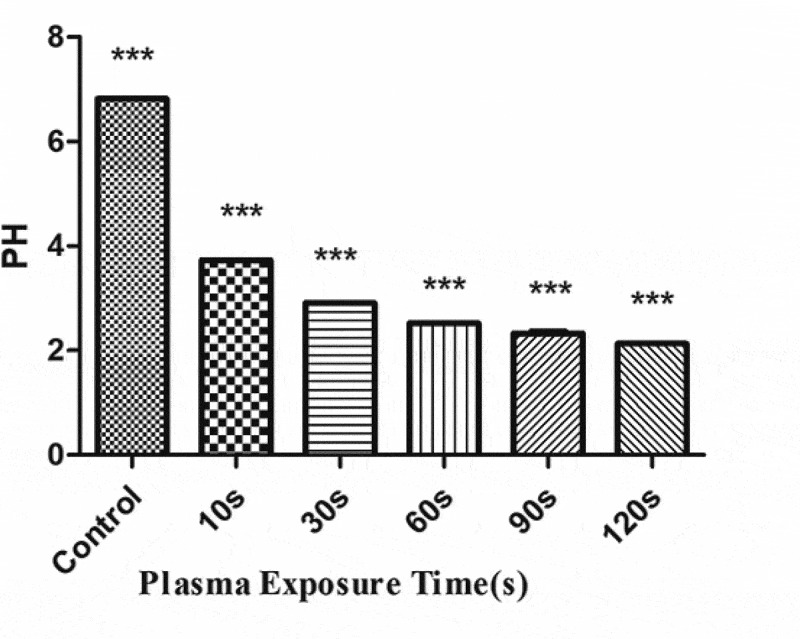
Changes in pH of the plasma-activated liquid treated for different periods (F = 16,340, *P* < 0.01)

The concentration of H_2_O_2_ (Figure 8) and NO_3_^−^ (Figure 9) gradually increased with the increase in the plasma treatment time, but the pH gradually decreased (Figure 10). The increased level of H_2_O_2_ and NO_3_^−^ and pH significantly correlated with the treatment time.

### Altered bacterial membrane integrity following PAL treatment

**Figure 11.**
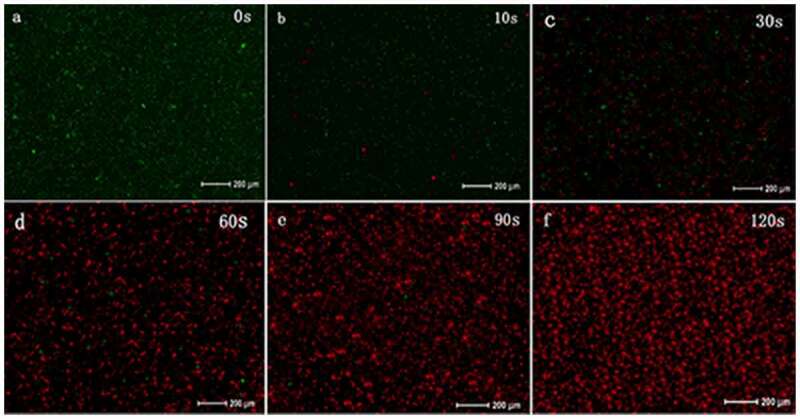
Changes in bacterial cell membrane integrity after treatment with plasma-activated liquid (PAL)

The LIVE/DEAD analysis (molecular probe) can distinguish cells with broken membranes based on the uptake of propidium iodide (Figure 11). When t* = 0 s, the proportion of dead bacteria (stained red) was less than 1%, and when t* = 10–30 s, the number of dead bacteria increased significantly. When t* = 90–120 s, the proportion of red-stained bacteria increased, and green-stained bacteria were rarely seen. As the PAL treatment time increased, the bacterial membrane integrity decreased.

### Structural changes in Ng after PAL treatment

**Figure 12.**
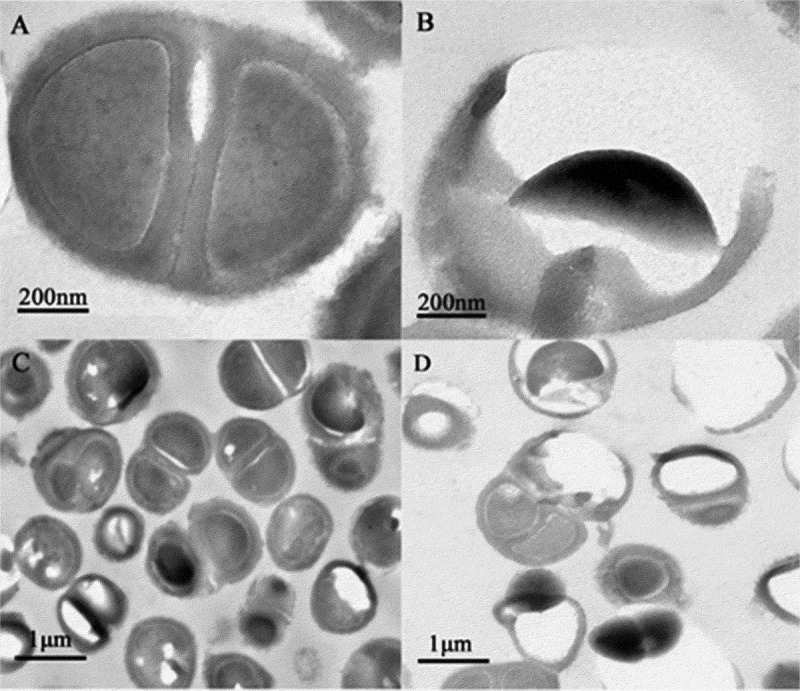
Changes in the bacterial structure photographed under an electron microscope. (a-d)**: a** and **c** represent the control group, and **b** and d represent the treatment group after plasma-activated liquid treatment

Ultrastructural changes in the bacteria were observed using a transmission electron microscope. *Ng* appeared as gram-negative diplococci under a high-powered microscope in the control group (Figure 12a). The cell structure was clear and complete, with a complete cell wall, thick flagella, and nuclear area (Figure 12a). Meanwhile, the treated group cells displayed ruptured and damaged cell walls, resulting in the release of cytoplasm, partial loss of cell walls, and outflow of nuclear contents (Figure 12b). Figure 12c shows the untreated bacteria, used as the control, observed under a low-magnification lens. Cells treated with PAL appeared ruptured, and the number of bacteria decreased in the same field of view (Figure 12d).

### Changes in gene expression in Ng after PAL treatment

**Figure 13.**
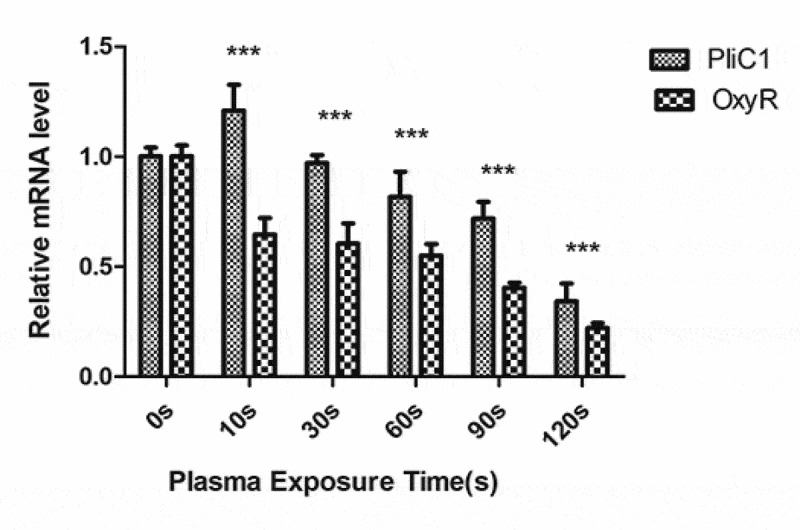
Expression of *PliC1* and *OxyR* in bacteria after plasma-activated liquid (PAL) treatment for different periods (F = 12.57, *P* = 0.002)

To understand the transcriptional changes in *PliC1* and *OxyR* expression following PAL treatment, their mRNA levels were measured using qPCR (Figure 13). In the control group, *PliC1* expression was set to 1. Compared with that in the control group, *PliC1* expression was upregulated to 1.22 in *Ng* after treatment for 10 s, and its transcription gradually decreased with the increase in the treatment time, ultimately decreasing to 0.34 at 120 s, which was significant (Figure 13). Similarly, *OxyR* transcription gradually decreased with the increase in the treatment time, and the ∆∆CT was 0.22 at 120 s (Figure 13).

### Analysis of IL-1β expression and IL-10/IL-17 factors in mouse splenocytes infected with Ng

#### *Expression of* IL-1β

**Figure 14.**
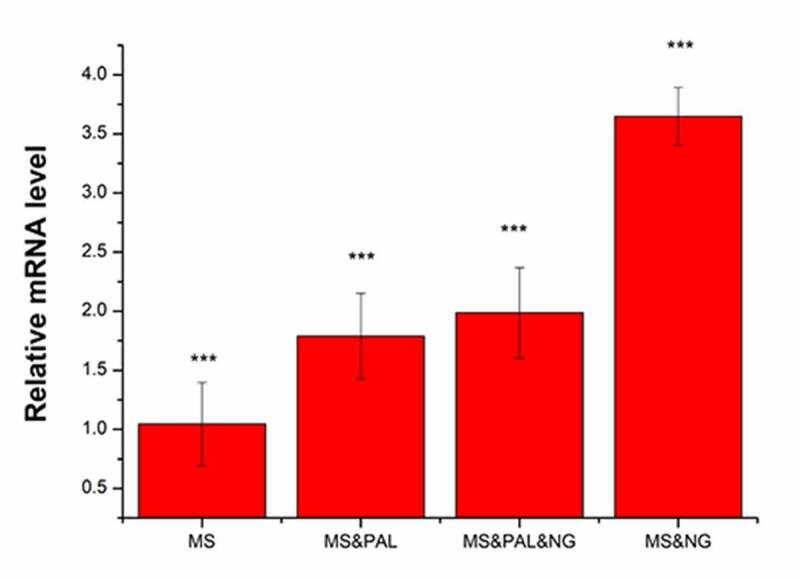
Expression of the inflammatory gene *IL-1β* in mouse splenocytes treated with *Neisseria gonorrhoeae* and plasma-activated liquid (PAL). MS, mouse splenocytes; NG, *Neisseria gonorrhoeae*; PAL, plasma-activated liquid

Mice were divided into four experimental groups as indicated in Figure 14. The expression of *IL-1β* was set to 1 in the MS group. In the MS & PAL group, the expression of *IL-1β* was upregulated to 1.79 compared with that in the MS group (*P = 0.039*). The expression of *IL-1β* increased to 2.39 in the MS & PAL & NG group; its expression in the MS & NG group was significantly higher than that in the other groups (*P*= 0.002).

#### Level of IL-10 and IL-17 in the culture supernatant of mouse splenocytes

**Figure 15.**
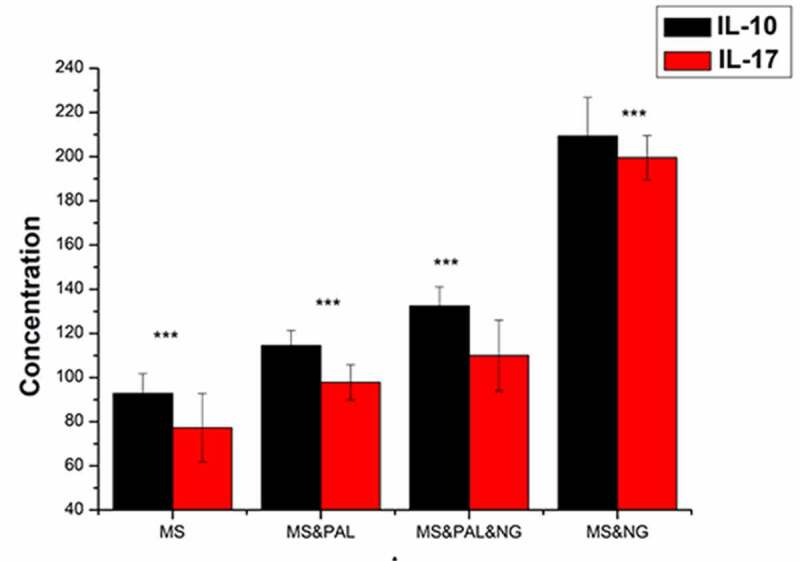
Expression of *IL-10* and *IL-17* in mouse splenocytes treated with *Neisseria gonorrhoeae* and plasma-activated liquid (PAL). MS, mouse splenocytes; NG, *Neisseria gonorrhoeae*; PAL, plasma-activated liquid

To assess the level of IL-10 and IL-17 in the culture supernatant of mouse splenocytes treated with *Ng* and PAL, the MS group was set as the blank group (Figure 15). The IL-10 and IL-17 levels in the cell culture supernatant of the MS & PAL group were higher than those of the MS group (IL-10, *P* = 0.0005; IL-17, *P* = 0.0005). Meanwhile, the levels of IL-17 and IL-10 were lower in the MS & PAL & NG group than in the MS & NG group (IL-10, *P* < 0.0001).

## Discussion

In the present study, no statistical differences were observed in the findings between the blank and control groups, the latter being immersed in physiological saline for 30 min. We excluded factors that affect the viability of bacteria, such as environment and immersion. However, the control group showed a statistically significant difference from the treated group, indicating that PAL exerts a bactericidal effect on *Ng*.

An analysis of PAL revealed that its major components are ROS and reactive nitrogen species (RNS), which can effectively inactivate various bacteria[16]. High concentrations of H_2_O_2_ (an ROS) are related to DNA, lipid, and protein damage[20]. Electrons, ions, and free radicals can also react with physiological saline (NaCl) to initiate chemical reactions to generate RS, including long-lived H_2_O_2_, H-, NO_3_, and O_3_ and short-lived OH^−^, O^−^, NO, ONOO^−^, and ClO_3_.

H_2_O_2_ is produced by the following reactions^−^:

*H_2_O +e^−^ → •H+•OH+e^−^*

*H_2_O + e^−^ → H_2_O^+^+ 2e^−^*

*H_2_O^+^+H_2_O → ˙OH + H_3_O*

H_2_O_2_ is generated primarily by the combination of •OH

$$OH+OH\to H_{2}O_{2}$$

$$O+H_{2}O\to H_{2}O_{2}$$

$$2H_{2}O+e^{-}\to H_{2}+H_{2}O_{2}+e^{-}$$

NO_3_ is generated from the following reactions:

$$N_{2}+e^{-}\to 2N+e^{-}$$

$$N_{2}+O\to NO+NO+O_{2}+M\to O_{3}$$

N+O→NO

$$2NO+H_{2}O_{2}+O_{3}\to HNO_{3}$$

$$N+O\to NO_{2}$$

$$NO+O_{3}\to NO_{2}+O_{2}$$

$$NO+HO_{2}\to HNO_{3}$$

$$NO_{2}+OH\to HNO_{3}$$

OH reacts with aqueous NaCl as follows:

$$OH_{\left(aq\right)}+Cl\to HPCl$$

$$HOCl^{-}+H^{+}\to H2O+Cl^{-}$$

$$Cl^{-}+Cl^{-}\to Cl_{2^{-}}$$

$$2Cl^{-}\to Cl_{\left(aq\right)}+Cl^{-}$$

$$Cl^{-}+H^{+}\to HCl$$

H^+^ can react with H_2_O_2_ in solution:

$$H^{+}+H_{2}O_{2}\to H_{2}O$$

However, owing to their short half-cycle and high reactivity, it is difficult to detect the concentration of short-lived RS in PAL. Therefore, only the long-lived RS, namely, H^+^, H_2_O_2_, and NO_3_^−^, were measured in PAL in this study. The pH and levels of H_2_O_2_ and NO_3_^−^ (RNS) increased with the increase in the plasma treatment time. H_2_O_2_ is related to the antibacterial properties of PAL[21], as it produces OH, which is a highly toxic ROS. These species are associated with several negative effects, including the loss of membrane potential and membrane integrity, peroxidation of membrane lipids, and changes in cellular proteins and DNA. Moreover, they can block DNA replication and transcription by causing single strand breaks to accumulate in cells [22–24].

Using the CFU method, we observed that the bactericidal effect elicited by PAL on *Ng* is dose- and time-dependent. Specifically, when the time of plasma treatment with physiological saline was fixed and the time of immersion of bacteria in PAL was prolonged, the number of bacteria that survived decreased. As the PAL treatment time increased, the levels of ROS and RNS generated increased, leading to an increase in bacterial death and resulting in dose-dependent effects. When the time of immersion of bacteria in PAL was increased, prolonged interaction of ROS and RNS with bacteria caused time-dependent effects. Although the CFU method is a common method for bacterial counting, it only allows the enumeration of surviving and dividing bacteria.

It has been reported that bacteria can demonstrate a viable-but-non-culturable (VBNC) status under natural stresses, such as starvation, extreme temperature, elevated osmotic pressure, and oxygen concentration [25–27]. As a mechanism of self-preservation, bacteria in this state can survive but lose the ability to divide and multiply to produce colonies. Reportedly, they also retain metabolic activity and show continuous gene expression, with the ability to recover under certain conditions [28–30].Therefore, if bacteria enter the VBNC state, the number of surviving bacteria counted using the classic plate colony counting method is lower than the actual number of surviving bacteria. Therefore, we combined the CFU method with bacterial viability test to detect metabolic activities in dormant bacteria that did not form colonies. The bacterial viability test demonstrated that after PAL treatment for 120 s, 10.15% of the bacteria continued to exhibit metabolic activity, whereas all bacteria were determined dead using the CFU method. This may partially explain the observed difference in the results between the detection methods following plasma treatment. These experiments showed that PAL can directly kill most *Ng* cells and the remaining bacteria enter the VBNC state. Under the PAL condition, it is difficult for bacteria to survive and reproduce, and the active substance (H_2_O_2_) in the plasma activation solution may be the main factor that induces *Ng* cells to enter the VBNC state [25,26].

The most common mechanism of ROS generation is the intracellular single-electron reduction of molecular oxygen leading to superoxide anion radical (O_2_^−^) formation; subsequently, superoxide dismutase converts superoxide to H_2_O[31]. When bacteria are immersed in PAL, exogenous ROS are produced, which damage the external structure of bacteria and penetrate the cells, resulting in increased ROS generation within the bacteria. However, at 60 s, the CFU counts showed that the number of bacteria decreased by 4.11 orders of magnitude, and the bacterial viability test results showed that the bacterial metabolic rate was approximately 59%. The principle of ROS detection relies on the oxidation of intracellular ROS by DCFH-DA to obtain fluorescent DCFH through enzymatic hydrolysis to generate fluorescence. As more cells die and the cell membrane is damaged, the more DCF probe remains unbound, which explains the decreasing trend of ROS levels observed after 30 s.

The membrane integrity and TEM analyses indicated that PAL disrupted the structures of the cell wall, cell membrane, and cell pili of bacteria. The adhesion of *Ng* to epithelial cells depends on the expression of PliC1, an outer membrane protein related to the fimbriae of *Ng*. After PAL treatment, *PliC1* expression initially increased and subsequently decreased. This transient increase may have been caused by the bacteria acting to combat oxidative stress in the body. For example, catalase in bacteria plays a vital role in protecting bacteria from ROS damage [32,33].Therefore, catalase can resist environmental changes within a certain range, but this ability is limited. As the dose of PAL increases, ROS in PAL can pass through the membrane and oxidize DNA or cause the peroxidation of active oxygen lipid, the final product of which may trigger oxidation and cause the subsequent DNA damage. Thus, as the dose of PAL increases, more bacteria die, causing a decreasing trend. Although there are several ROS regulatory systems in bacteria, the OxyR regulatory system is particularly important[34]. As the treatment time gradually increased, the *OxyR* expression gradually decreased. The initial decline was found to be relatively slow, which may regulate ROS and help the bacteria to adapt to the mutant environment that is not conducive to survival. However, in the later stages, owing to the massive destruction of bacteria, the expression level of the gene declined.

The activation of NLRP3-mediated inflammatory response pathways is a major factor associated with the host response and pathogenesis of *Ng*. It plays a role in exogenous microorganisms or endogenous danger signal receptors, regulates the maturation and secretion of inflammatory cytokines such as IL-10 and IL-17, and participates in natural and acquired immunity[35]. Here, the *in vitro* experiment with mouse spleen cells infected with *Ng* was performed to explore the body’s immune regulation against *Ng* infection and increased inflammatory gene expression and inflammatory factor release following PAL intervention, thereby promoting disease alleviation. Mouse splenocytes infected with *Ng* showed upregulated expression of inflammatory factors to combat such an infection. After splenocyte treatment with PAL, the active substances in PAL stimulated the release of inflammatory factors, which was not observed in the control group. Previous studies have shown that PAL exerts an effective bactericidal effect. [16,18]The concentration of bacteria affects the release of inflammatory factors[36]. Therefore, here, after the addition of PAL, the bacterial concentration in the infected group reduced, resulting in lower expression of inflammatory genes and factors than that in the infected group. PAL can simultaneously stimulate the expression of inflammatory genes, release of inflammatory factors, and immune status against *Ng* infection to a certain extent. As *Ng* primarily infects mucosal tissues, PAL can be administered as an emulsion for clinical surface infections, such as vaginal infections, neonatal mucositis, and other body surface infections, with good results. Therefore, PAL use represents a new treatment method to replace antibiotics; it can also be used in combination with antibiotics. Especially for neonatal gonococcal conjunctivitis, PAL prepared with physiological saline can simulate tears. Furthermore, the solution has good biocompatibility, has no toxic effects on living cells or tissues, and causes little eye irritation. It has been reported that PAL has no killing effect on keratinocytes in vitro[37].However, certain challenges remain in the practical application of PAL. First, there is no quantitative standard to treat different individuals with PAL, and randomized controlled clinical trials are needed to verify the standardized treatment for specific infections. Second, the negative effects of the active ingredients present in PAL must also be considered.

## Conclusions

PAL was observed to exert an antibacterial effect on *Ng*. After PAL treatment, the bacteria were destroyed, as observed by CFU counts and the bacterial viability test. Furthermore, PAL immersion induced morphological changes, including disruption of the bacterial membrane and depletion of fimbriae. Long-lived RS ions in PAL destroy the cell membrane and disrupt ROS balance in cells by penetrating the cells, which ultimately hinders bacterial metabolism, leading to bacterial death. Furthermore, PAL was found to promote the expression of inflammatory genes and the release of inflammatory factors, to a certain extent, during the sterilization process in *Ng-*infected mouse spleen cells. The results of this study provide a theoretical basis for further research on the clinical application of PAL.

## Highlights

- PAL was observed to exert an antibacterial effect on *Ng*.
- PAL immersion disrupted the morphological structure of bacteria.
- PAL upregulated inflammatory gene expression in *Ng-*infected mouse spleen cells.
- PAL may affect the immune status of the host during infection.
- This study provides theoretical basis for clinical application of PAL.
